# Traumatic Lung Herniation following Skateboard Fall

**DOI:** 10.1155/2016/9473906

**Published:** 2016-10-30

**Authors:** Dafney L. Davare, Chauniqua Kiffin, Rafael Sanchez, Seong K. Lee, Eddy H. Carrillo, Andrew A. Rosenthal

**Affiliations:** Division of Acute Care Surgery and Trauma, Memorial Regional Hospital, 3501 Johnson Street, Hollywood, FL 33021, USA

## Abstract

Lung herniation (LH) is a rare clinical entity involving the protrusion of lung outside the thoracic cage. It has a variety of etiologies and clinical presentations, making diagnosis difficult. We present a case of a 20-year-old male who reported pleuritic pain after falling from a skateboard. Evaluation through computed tomography (CT) scanning of the chest revealed an anterior lung hernia associated with rib fractures. This case emphasizes the need for clinicians to include lung herniation in the differential diagnosis of patients with trauma and inexplicable or persistent pulmonary issues.

## 1. Introduction

Lung herniation (LH) is the migration of pulmonary tissue outside the thoracic cage [1–4]. It is an extremely rare occurrence with an unknown prevalence and incidence [5, 6]. The clinical presentation of LH is often nonspecific and can mimic the symptoms seen in other pulmonary disease processes. In addition, the cause of LH varies greatly. Due to its rarity, LH management standards have not been established. As such, treatment in case reports has ranged from conservative nonoperative management to surgical reduction and chest wall reconstruction [3, 7]. For the clinician encountering LH, dilemmas in determining the appropriate treatment option can arise. We present a case of LH following blunt force trauma.

## 2. Case Report

**Figure 1:**
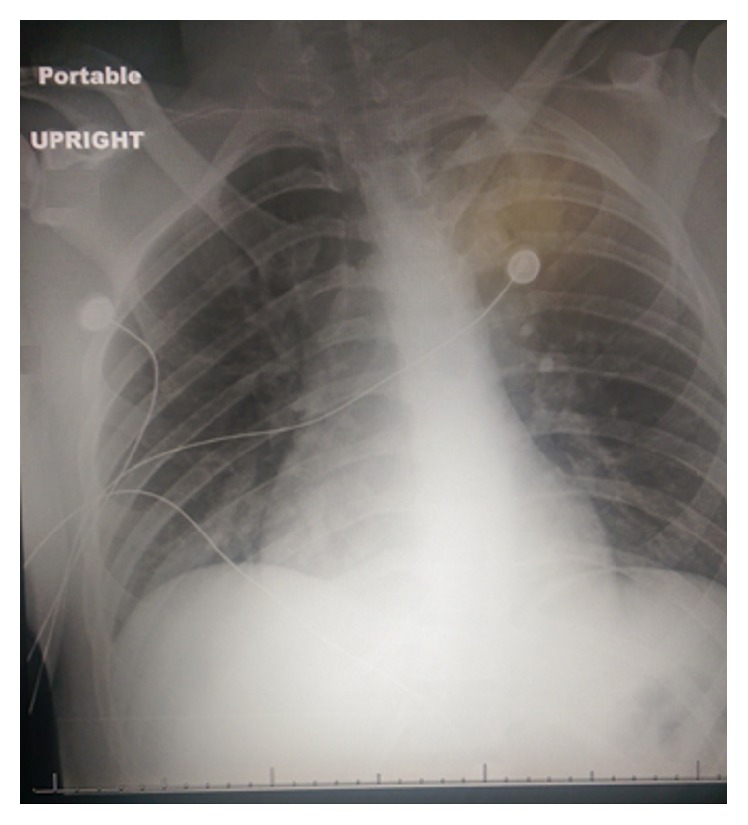
Anterior-posterior chest radiograph showing no obvious abnormalities in the thoracic cavity.

**Figure 2:**
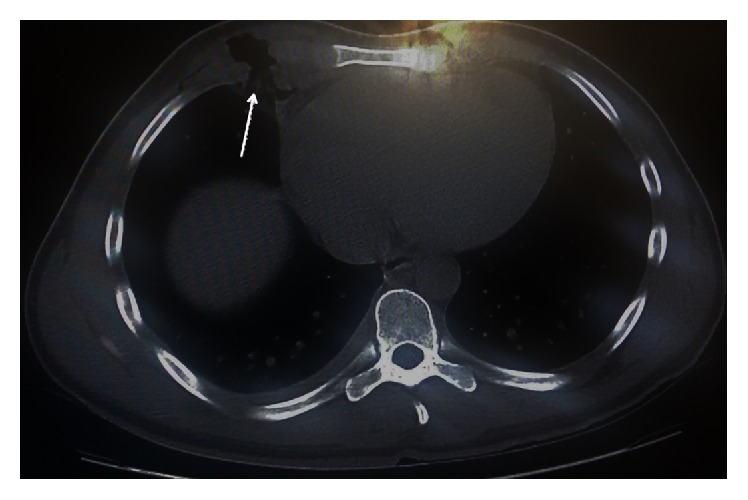
Axial contrast-enhanced CT of the chest. Note the disruption in the thoracic cavity with lung anterior herniation due to fractured ribs (arrow).

A 20-year-old healthy male presented to the emergency department after falling from a skateboard. He sustained blunt trauma to his right chest wall, resulting in pleuritic pain and mild dyspnea. Diagnostic workup included chest radiography (CXR), which showed no acute findings (Figure 1). Further evaluation with chest computed tomography (CT) scan demonstrated fractures of the right 4th through 6th ribs. Herniation of the middle lobe of the right lung was also identified on CT scan (Figure 2).

**Figure 3:**
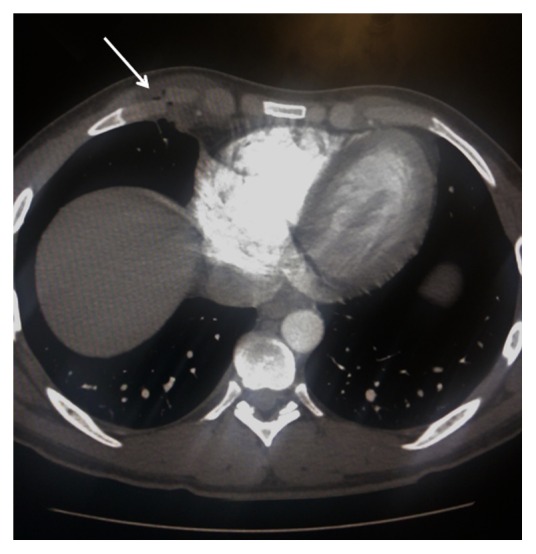
Axial contrast-enhanced CT 10 days after injury. Note the improvement in lung herniation (arrow).

The patient was admitted for observation and pain management. His pain and dyspnea improved and he was managed conservatively. A repeat CXR on hospital day two was stable, and the patient was discharged home. Ten days after discharge a follow-up chest CT scan was completed and demonstrated a decrease in herniated lung (Figure 3).

## 3. Discussion

Lung herniation is a rare entity that is seldom life-threatening. According to Morel-Lavallee, LH can be classified based on etiology and location [1, 5, 8]. Locations include cervical, thoracic, and diaphragmatic regions. Lung herniation is most commonly encountered through the anterior thoracic cage [1–3, 5]. Anterior intercostal or parasternal types are the two most common types of thoracic LHs; anterior intercostal hernias account for over 98% of LHs [1, 2, 9]. Posterior thoracic LHs are uncommon as musculature of the back provides reinforcement to the posterior thoracic cage [5, 7]. Cervical and diaphragmatic LHs are very rare with few cases reported in literature.

Etiology of LH can be classified into congenital and acquired type [1, 7, 8]. Congenital causes are less common and related to connective tissue disorders or abnormal chest wall development [10]. Ehlers-Danlos, for example, is one such connective tissue disorder that results in tissue laxity and fragility [11]. In these patients there is a greater risk of lung herniation due to the inherently weaker tissue. There has also been a case report on an otherwise healthy young male patient who was found to have an incidental lung herniation due to congenital malformation of his chest wall [6].

Acquired LH is the most common etiology of pulmonary protrusion outside the thoracic cage [9, 12]. It is seen in patients with significant blunt chest trauma, as well as penetrating chest injuries, and thoracic surgical procedures [12]. As discussed, anterior intercostal LH location is the most frequently encountered location and is consistent with LH's association with traumatic rib fractures.

It is important to recognize that the development of LH requires a combination of anatomical and physiological factors. To promote migration of lung tissue outside its usual domain there must be weakness or defect in the structural boundaries and sudden or chronic increase in intrathoracic pressure [3, 13]. For cervical herniations, a defect in Sibson's fascia is identified [1]. For thoracic herniation there is separation or fracture of ribs with weakening of the intercostal muscles [2, 7, 9]. The eventual herniation of lung through these weakened areas occurs when intrathoracic pressure increases as seen in chronic cough or significant chest impact. Patients with chronic obstructive pulmonary disease (COPD) have classic physiological and anatomical changes that support the development of spontaneous thoracic LHs. In these patients, weakened thoracic cages are often due to chronic steroid use, and promotion of LH is seen with chronic coughing and hyperinflated lungs [3, 6, 13, 14].

The clinical presentation of LH varies. It is important to include this in the differential diagnosis of patients with a history of chest trauma, thoracic surgery, or persistent pulmonary symptoms. Some patients with LH present with a crepitant chest wall mass that worsens with Valsalva and improves with normal breathing [6, 8, 9, 13]. However, most cases of LH present with nonspecific symptoms such as cough and dyspnea, which suggests LH may be underdiagnosed [5, 6]. As discussed earlier, LH is rarely life-threatening, but serious complications have been reported including lung tissue strangulation, lung necrosis, pneumothorax, and pneumomediastinum [3, 5–7, 9]. In one case report, a patient developed multiorgan failure after LH resulted in respiratory failure [13]. Suspicion of LH warrants diagnostic imaging. Chest radiography (CXR) is often the initial diagnostic test. It is imperative that the clinician obtain both lateral and posteroanterior views, as the posteroanterior view alone may be falsely negative. Lightwood and Cleland suggest obtaining CXR “during forced expiration against a closed glottis” [1].

Another diagnostic option is ultrasonography. In trauma, patients are evaluated with the eFAST or extended focus assessment with sonography in trauma. This rapid diagnostic test involves assessment of the pleural cavities to identify pneumothoraces [15]. In one reported case the eFAST assisted physicians in identifying a trauma-related LH [15].

Computed tomography (CT) scan of the chest is the most sensitive diagnostic exam for LH. It provides excellent characterization of LH [2–4]. It is also valuable in identifying associated complications and aids in the surgical planning of LH repair [3, 4].

When a patient is diagnosed with LH, it is paramount that the etiology, location, and associated symptoms are assessed and evaluated to determine treatment. The management of LH depends primarily on its associated symptoms. Large lung hernias creating physical deformities, presence of lung tissue necrosis, recurrent infections, pain, and failure of symptoms to improve with conservative management warrant operative intervention [3, 9]. This involves reduction of the herniated tissue, possible resection of necrotic tissue, and repair of the associated thoracic cage defect with autologous tissue or synthetic mesh [3, 5, 6, 10]. Those patients who are asymptomatic with smaller defects can be managed conservatively, with close monitoring [5–7, 13].

Although LH is rare and usually benign, it is imperative to evaluate this injury any time a patient has sustained trauma to the chest wall. Any chest wall trauma, defect, or thoracic surgical intervention may allow lung tissue to protrude into the thoracic cavity. Patients with these histories who worsen or do not clinically improve will require evaluation for LH and surgical management. Those who are diagnosed with small asymtomatic lung hernias can be closely followed up but may require surigcal intervention if the LH enlarges or symtoms develop. Therefore, early consultation with a trauma or thoracic surgeon upon diagnosis of LH is recommended.
